# Synergistic Pre-Oxidation and CVD Engineering for Precise Closed-Pore Construction in Coffee Grounds-Derived Hard Carbon Anodes for High-Performance Sodium-Ion Batteries

**DOI:** 10.3390/ma19122495

**Published:** 2026-06-10

**Authors:** Xinjie Sun, Hui Yang

**Affiliations:** College of Materials Science and Engineering, Nanjing Tech University, Nanjing 211816, China

**Keywords:** sodium-ion battery, hard carbon anode, coffee grounds, pre-oxidation, chemical vapor deposition (CVD), closed pore engineering

## Abstract

Upcycling biomass waste into value-added battery materials is crucial for sustainable energy storage. Here, we transform coffee grounds into high-performance hard carbon (HC) anodes for sodium-ion batteries (SIBs) via a synergistic pre-oxidation and acetylene chemical vapor deposition (CVD) strategy, which effectively reduces open pores and promotes structural stabilization. The resulting material exhibits features consistent with a closed-pore architecture. Pre-oxidation incorporates oxygen-containing functional groups that template accessible pores and expand the interlayer spacing during carbonization. Subsequent CVD covers surface pores and contributes to the stabilization of the pore structure. The optimized HC (COF300&1300@C) exhibits a balanced set of structural features, including a low specific surface area (2.1 m^2^ g^−1^), expanded interlayer distance (0.391 nm), and a well-regulated pore system with reduced surface area and controlled pore size. As a result, it delivers a reversible capacity of 298 mAh g^−1^ with an ICE of 70%, and remarkable cycling stability (97% capacity retention after 500 cycles at 1C). This study elucidates the synergistic mechanism of pre-oxidation and CVD in reducing open pores and stabilizing the pore architecture, thereby yielding characteristics indicative of closed-pore behavior, and providing a novel and efficient approach for designing high-performance biomass-derived hard carbons for energy storage.

## 1. Introduction

The escalating demand for large-scale energy storage systems has spurred the search for alternatives to lithium-ion batteries (LIBs) owing to cost and resource concerns [1,2,3]. Sodium-ion batteries (SIBs), with similar electrochemistry to LIBs [4,5,6], are promising due to low cost and natural abundance [7,8]. Developing high-performance anodes is essential for SIBs. While candidates such as metal oxides/sulfides [9,10], organics [11,12], and alloys [13,14], have been explored, they often suffer from poor conductivity, limited cycle stability and significant volume expansion. In contrast, hard carbon (HC) is considered to be one of the most promising anode materials for SIBs due to its larger interlayer spacing than graphite, which facilitates Na^+^ insertion, and its high theoretical specific capacity of 530 mAh g^−1^ arising from multiple storage sites including interlayers, defects, and nanopores [15,16]. HC consists of curved graphene nanosheets and turbostratic domains with enlarged interlayer spacing, together with micropores formed among these microstructural features [17,18], making a complex sodium storage mechanism. To date, researchers have proposed four sodium storage mechanisms [19,20]: “intercalation-adsorption” model, “adsorption-intercalation” model, “adsorption-intercalation-filling” model, and “adsorption-filling” model. The storage mechanism remains controversial due to HC’s complex structure [21,22].

Biomass-derived HC is promising as anode material owing to the easy availability of raw materials and eco-friendliness during preparation. Nevertheless, biomass-derived HC inherently suffers from low initial coulombic efficiency (ICE), limited reversible capacity and poor C-rate performance, which can be attributed to excessive open pores leading to electrolyte decomposition, abundant surface defects, and the lack of a well-regulated pore architecture. To achieve hard carbon with high ICE, high reversible capacity, and excellent C-rate performance, the material should possess a low specific surface area to minimize electrolyte side reactions, a suitable density of structural defects to provide additional Na^+^ adsorption sites, and a regulated pore architecture with fewer open pores but sufficient closed pores to provide stable Na^+^ storage sites. Nowadays, researchers have proposed three modification strategies, including heteroatom doping [23,24], pore regulation [25,26] and functional group introduction [27]. Heteroatom doping (e.g., N, P, S) can expand interlayer distance, promote conductivity, and introduce defect sites that serve as additional sodium storage centers, although excessive defects may also trigger side reactions and reduce ICE [28,29,30]. For instance, Sun et al. [31] synthesized N-doped HC with enlarged interlayer spacing (0.38 nm) from natural silk, showing an ultra-stable capacity of 161.5 mAh g^−1^ at 2 A g^−1^ for over 5000 cycles. The C–N bonds disrupt the ordered stacking of carbon layers, weaken the interlayer van der Waals forces, and thereby enlarge the interlayer spacing. The graphitic N-doping introduced extra electrons, thus improving the electrical conductivity. Meanwhile, more defects were generated by the introduction of pyridinic N (located at the edge of the carbon hexagonal ring, replacing carbon atoms to form defect edge sites). Pore structure is critical. Open pores are highly accessible to electrolytes, facilitating adsorption and chemical reactions, and thereby promoting fast Na^+^ transport and improving sloping capacity. However, their pore surface accelerates upon exposure to electrolyte, leading to capacity loss and reduced ICE. Closed pores provide stable plateau capacity and maintain carbon matrix integrity, thereby reducing irreversible capacity loss and enhancing cycling stability [32,33,34]. Therefore, achieving an optimal balance between open and closed pores is key for high energy density and coulombic efficiency. Zheng et al. [35] successfully prepared starch-derived HC with abundant closed pores by combining CO_2_ etching with high-temperature carbonization, showing high ICE (91%) and plateau capacity (351 mAh g^−1^), as well as excellent cycle performance (96% retention after 100 cycles at 200 mA g^−1^). The existence of functional groups (e.g., via oxygen-containing functional groups) can provide additional adsorption sites through coordination interactions with Na^+^ [36]. Lou et al. converted C=O into C–O by hydrogen reduction treatment in red willow, thereby increasing the number of Na^+^ adsorption sites and enhancing the reversible capacity. The resulting HC exhibited a capacity of 325 mAh g^−1^ with an ICE of 80.84% [37].

However, most researchers currently focus on a single modification method, and few combine two or all three of the aforementioned modification methods. Therefore, using coffee grounds as the hard carbon precursor, and combining functional group introduction with pore structure regulation, we propose a modification strategy that combines pre-oxidation with acetylene chemical vapor deposition to enhance the electrochemical performance of the hard carbon. During the pre-oxidation process, oxygen-containing functional groups are introduced onto the surface of the hard carbon. During subsequent high-temperature carbonization, these groups decompose to release CO/CO_2_, inducing localized bending of the carbon layer and increasing the interlayer spacing, thereby enhancing the plateau capacity. At the same time, the locally bent carbon layer tends to form closed pores, further increasing the plateau capacity. Subsequent acetylene CVD treatment modifies HC in three distinct aspects: firstly, sp^3^-C with C–H bonds and weakly stable oxygen-containing functional groups are more prone to bond breaking, leading to the formation of dangling bonds related to free radicals, thus inducing inherent defects such as edges and enhancing the sloping capacity; secondly, defects such as pentagons and heptagons disrupt the integrity of the sp^2^-C conjugated network growth and lead to the bending of carbon layers, and consequently enlarge the interlayer spacing and enhance the plateau capacity; and thirdly, acetylene pyrolytic carbon can form a uniform coating layer on the surface of HC, converting open pores into closed pores. This process increases the number of closed pores while reducing the specific surface area (SSA), ultimately enhancing the plateau capacity and ICE. Through comprehensive structural and electrochemical analysis, we demonstrate that this strategy combines functional group introduction with pore regulation, and their synergistic effects significantly enhance the ICE, rate performance, and reversible capacity of coffee grounds-derived HC, offering a promising route for value-added utilization of biomass waste.

## 2. Experimental

### 2.1. HC Synthesis

**Figure 1:**
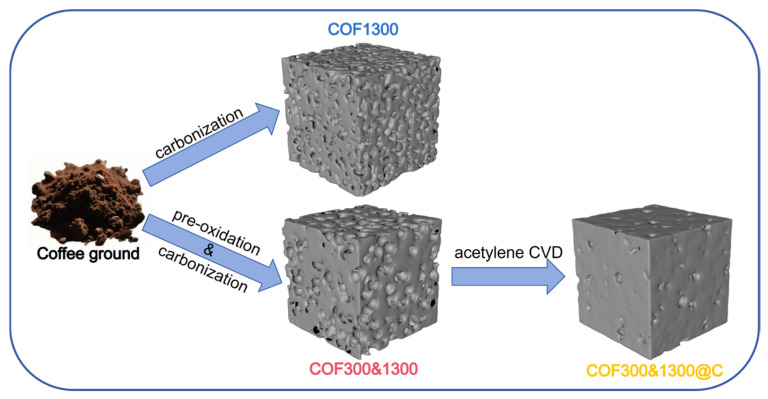
Schematic illustration of the synergistic pre-oxidation and CVD strategy for engineering the pore structure in coffee grounds-derived HC.

The schematic representation of the HC preparation process is provided in Figure 1. First, the coffee grounds were placed in a dry oven to remove moisture. Then, they were ground and sieved through a 200-mesh screen, and heated at a rate of 5 °C/min^−1^ in an air atmosphere to 300 °C and held at this temperature for 2 h. After cooling to room temperature, they were heated in an inert atmosphere (N_2_) at a rate of 5 °C/min^−1^ to 1300 °C and maintained at this temperature for 4 h. After cooling to room temperature once more, the HC material was acid-soaked with a 2 mol L^−1^ HCl aqueous solution at room temperature for several hours, filtered, thoroughly washed with deionized water, and then dried in an oven at 80 °C for 12 h. Finally, the dried material was heated at a rate of 5 °C/min^−1^ to 700 °C in a mixed atmosphere of N_2_ and C_2_H_2_ (N_2_: 50 sccm; C_2_H_2_: 10 sccm), and held at this temperature for 2 h. The resulting HC material, cooled to room temperature, was designated as COF300&1300@C. For comparison, two control samples were synthesized: COF1300, which is HC obtained via one-step high-temperature sintering without pre-oxidation and CVD coating, and COF300&1300, which is HC produced through a two-step sintering route with pre-oxidation but without CVD carbon coating.

### 2.2. Physicochemical Characterization

The functional groups of the HC precursors were characterized by Fourier-transform infrared spectroscopy (FTIR, Bruker Tensor II, Ettlingen, Germany) in the range of 4000–500 cm^−1^. The structural parameters of the HC was analyzed using X-ray diffraction (XRD, Rigaku SmartLab SE diffractometer, Cu Kα, Tokyo, Japan) within a range of 10–80°. The morphology of the HC was examined using scanning electron microscopy (SEM, JEOL, JSM-7600F, Tokyo, Japan) and transmission electron microscopy (TEM, JEOL JEM-2100, Tokyo, Japan), whereas elemental composition was analyzed with energy-dispersive spectrometry (EDS). The graphitization degree of HC was analyzed by Raman spectroscopy (Raman, Horiba LabRAM HR Evolution, Palaiseau‌, France) with a 532 nm excitation laser. The specific surface area and pore size distribution of HC materials were characterized through N_2_ adsorption–desorption isotherms (Micromeritics ASAP 2460, Norcross, GA, USA). The elemental state of the elements on the surface was analyzed by X-ray photoelectron spectroscopy (XPS, Thermo Scientific K-Alpha, Waltham, MA, USA).

### 2.3. Electrochemical Characterization

A total of 160 mg of HC material was dissolved in 2.5 mL N-methyl-2-pyrrolidone (NMP) with 20 mg PVDF and 20 mg super P. After being stirred for 6 h, the slurry was evenly coated on the copper foil (loading of active material: ~1–2 mg cm^−2^), and then dried in a vacuum oven at 70 °C for 12 h. After the drying process was completed, it was cut into 13 mm electrode slices, which were paired with Na metal electrodes and glass fiber separator. The electrolyte is NaClO_4_ (1 M NaClO_4_ in EC:PC = 1:1 Vol% with 5% FEC). The components described above were assembled into half-cells inside an argon-filled glove box. Electrochemical performance was evaluated using a Neware battery tester: the cells were charged/discharged at a current density of 10 mA g^−1^ for three cycles between 0.01 and 3 V, followed by cycling at current densities from 30 mA g^−1^ (0.1 C, 1 C = 300 mA g^−1^) to 300 mA g^−1^ for long-term cycling and rate capability (C-Rate) tests. Furthermore, to investigate the electrochemical behavior, cyclic voltammetry (CV), electrochemical impedance spectroscopy (EIS), and galvanostatic intermittent titration technique (GITT) measurements were performed on the cells using a Land battery testing system.

## 3. Results and Discussion

**Figure 2:**
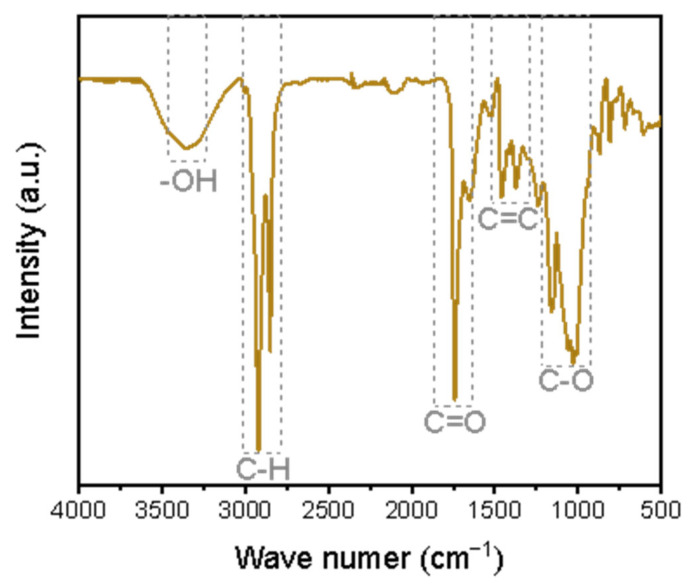
Fourier-transform infrared spectra of coffee grounds.

Here, coffee grounds were chosen as the precursor for HC synthesis. The first reason is that the approximately 18 million tons of coffee grounds produced globally each year release methane, which contributes to global warming; reusing coffee grounds can help alleviate this problem and support sustainable development. Secondly, coffee grounds inherently contain abundant oxygen-containing functional groups and aliphatic carbon chains, shown in Figure 2, facilitating the formation of the micropores/mesopores and disordered carbon structures required for HC.

In this study, 300 °C was selected as the pre-oxidation temperature, for the following reasons. Firstly, the temperature range of 200–400 °C is where thermolysis and cross-linking reactions occur in the lignin and cellulose of coffee grounds. Secondly, with 300 °C as the midpoint of this range, this temperature ensures cross-linking between oxygen-containing functional groups and the carbon backbone while preventing quality loss caused by excessively high temperatures [38].

### 3.1. Morphological and Structural Characterization of HC Under Synergistic Treatment

**Figure 3:**
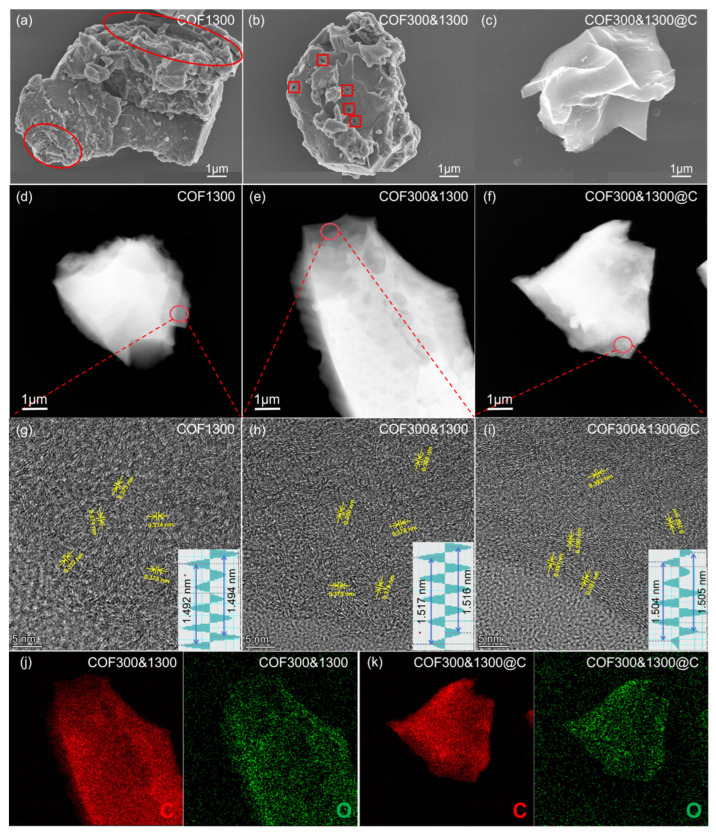
Morphology and microstructure: (**a**–**c**) High-magnification SEM images; (**d**–**f**) TEM images; (**g**–**i**) HRTEM images with measured interlayer spacings; (**j**,**k**) EDS elemental mappings for COF300&1300 and COF300&1300@C.

SEM and TEM were employed to examine the effects of pre-oxidation and carbon coating on the morphology and structure of the coffee grounds-derived HC (Figure 3 and Figure S1). Low-magnification SEM images (Figure S1) reveal that all materials, COF1300, COF300&1300, and COF300&1300@C, display irregular block-like morphologies with particle sizes in the range of 1–10 μm. The particles of the pre-oxidized and carbon-coated samples are significantly larger than that of COF1300. High-magnification SEM images (Figure 3a–c) present the morphology of HCs. COF1300 shows a rough and wrinkled surface, potentially contributing to an increased SSA. Pre-oxidation (COF300&1300) results in a smoother surface with more uniform and smaller pores (approximately 200 nm). The subsequent CVD treatment (COF300&1300@C) produces a remarkably smooth surface with layered contours and no visible pores, suggesting effective pore sealing by carbon layer deposition. TEM and HRTEM images (Figure 3d–i) confirm the typical disordered structure of HC. Crucially, HRTEM reveals a progressive expansion of the (002) interlayer spacing (d_002_) from 0.373 nm (COF1300) to 0.379 nm (COF300&1300) and finally to 0.391 nm (COF300&1300@C) (Figure 3g–i). First, during the pre-oxidation process, oxygen-containing functional groups are introduced onto the surface of the hard carbon. During the subsequent high-temperature carbonization process, these groups decompose to release CO/CO_2_, inducing localized bending of the carbon layer, thereby increasing the interlayer spacing [39]. Second, during CVD treatment, carbon radicals form covalent, sp^3^-hybridized linkages with the carbon matrix. This process disrupts the in-plane conjugated structure, causes lattice distortion, weakens interlayer van der Waals forces, and consequently leads to further spacing expansion, which is highly beneficial for Na^+^ intercalation [40,41]. Finally, EDS mapping (Figure 3j,k) confirms a uniform distribution of C and O on the particle surface for COF300&1300 and COF300&1300@C.

**Figure 4:**
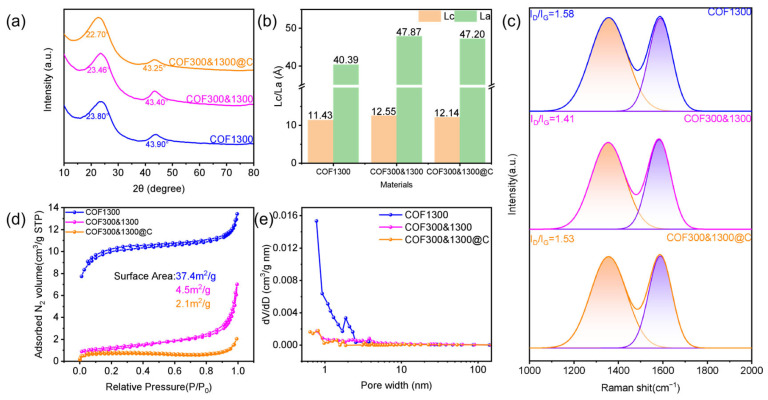
Structural and textural characterization: (**a**) XRD patterns, (**b**) calculated crystallite sizes *L_c_* and *L_a_*, (**c**) Raman spectra, (**d**) N_2_ adsorption-desorption isotherms, and (**e**) pore size distributions.

The structural parameters of the HC were further investigated using XRD and Raman spectroscopy. The XRD patterns (Figure 4a) show broadened (002) and (100) peaks, confirming that they all have a typical highly disordered nature with graphite microcrystals. The interlayer spacing d_(002)_ values calculated from the Bragg equation for COF300&1300 and COF300&1300@C are 3.79 Å and 3.91 Å, respectively, both larger than that of COF1300 (3.73 Å), consistent with the HRTEM observations. The average structural domain sizes along the c-axis (*L_c_*) and a-axis (*L_a_*) were derived using Scherrer formula [42]. Both *L_c_* and *L_a_* reach maximum values for COF300&1300 (*L_c_* = 11.94 Å, and *L_a_* = 20.24 Å), indicating that the pre-oxidation strategy promotes localized aromatization and ordered domain growth, as can be seen in the HRTEM images. After carbon coating (COF300&1300@C), *L_c_* and *L_a_* decrease slightly (10.12 Å and 18.62 Å, respectively), which may reflect the influence of the carbon layer deposited on the surface and pore structure, leading to a reduction in apparent ordered domain sizes. Raman spectra (Figure 4c) display characteristic D (~1350 cm^−1^) and G (~1580 cm^−1^) bands, corresponding to disordered/defective sp^2^ carbon and ordered graphitic domains, respectively [43]. The intensity ratio I_D_/I_G_ decreases from 1.58 (COF1300) to 1.41 (COF300&1300), indicating that pre-oxidation introduces oxygen-containing functional groups which act as templates for pore regulation and promote more uniform carbonization, thereby reducing structural disorder and defect density. A further rebound to 1.53 for COF300&1300@C can be attributed to the pyrolysis of acetylene generating a thin carbon layer that seals open pores but simultaneously produces unstable sp^3^-C sites prone to cleavage, leading to the formation of edge defects. Figure 4d,e presents the N_2_ adsorption–desorption isotherms together with the pore size distributions of the prepared HC materials. All isotherms are type IV, indicating the presence of both micropores and mesopores. The SSA calculated by the BET method decreases markedly from 37.4 m^2^ g^−1^ for COF1300 to 4.5 m^2^ g^−1^ for COF300&1300, owing to surface smoothing and pore regulation during pre-oxidation. After carbon coating, SSA further drops to 2.1 m^2^/g^−1^, as the deposited carbon layer effectively covers open pores. Total pore volume follows a similar trend, declining from 0.0208 cm^3^/g^−1^ (COF1300) to 0.0108 cm^3^/g^−1^ (COF300&1300) and finally to 0.0032 cm^3^/g^−1^ (COF300&1300@C). Pore-size distribution by BJH and t-plot models reveal that pre-oxidation and CVD treatment transform the majority of open pores into closed pores and further narrow the pore sizes. In summary, pre-oxidation promotes structural ordering and enlarges interlayer spacing, while the carbon coating further expands the d-spacing, and significantly lowers SSA by pore filling, collectively optimizing the HC for Na^+^ storage.

**Figure 5:**
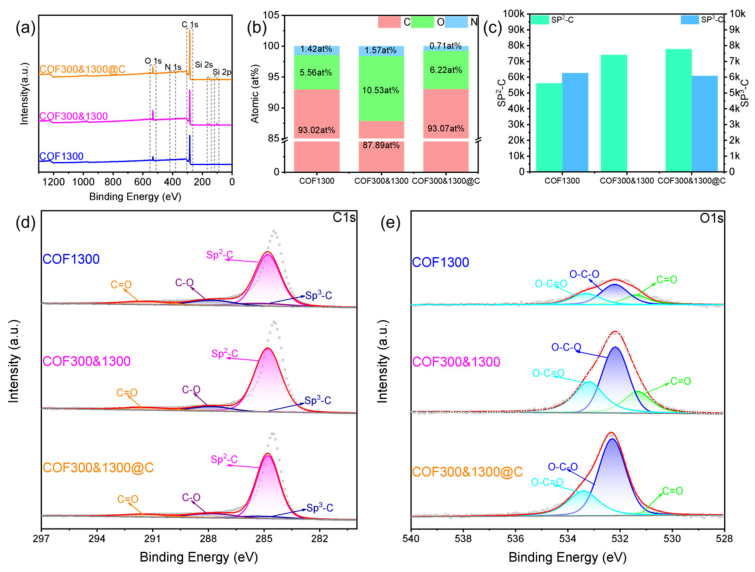
Analysis of chemical state of elements on the surface: (**a**) XPS survey spectra, (**b**) atomic percentages of C, O, and N, (**c**) Sp^3^/Sp^2^ carbon ratio from C 1s fitting, (**d**) high resolution C 1s XPS peaks, and (**e**) high resolution O 1s spectra.

In addition, XPS analysis was employed to investigate the effects of pre-oxidation and carbon coating treatments on the chemical state of elements on the hard carbon surface. The survey spectra (Figure 5a) confirm the presence of C 1s (284.8 eV) and O 1s (532.0 eV) signals for all samples, along with negligible N and Si signals. The atomic percentages (Figure 5b) reveal significant changes induced by the treatments. Pre-oxidation (COF300&1300) increases the oxygen content to 10.53 at% and reduces the carbon content to 87.89 at%, due to carbon loss as CO/CO_2_ and the introduction of oxygen-containing functional groups. After carbon coating (COF300&1300@C), the carbon content rises to 93.07 at% while oxygen decreases to 6.22 at%. This change mainly reflects the contribution of the deposited carbon layer, which increases the overall carbon fraction and thereby lowers the relative oxygen content. High-resolution C 1s spectra (Figure 5d) were deconvoluted into SP^2^–C (284.8 eV), SP^3^–C (285.2 eV), C–O (287.8 eV), and C=O (291.5 eV) components. The change of Sp^2^–C and Sp^3^–C content is summarized in Figure 5c. Sp^2^–C exhibits a sustained upward trend, while Sp^3^–C shows a pattern of initially declining followed by a rebound. The Sp^2^–C fraction shows a continuous increase, whereas Sp^3^–C first decreases after pre-oxidation and then recovers slightly after coating. This trend is attributed to the stabilization and expansion of aromatic structures during pre-oxidation, which increases Sp^2^–C content, while the more reactive Sp^3^–C bonds are preferentially consumed [44]. The subsequent CVD process deposits additional Sp^2^–C-dominated amorphous carbon, leading to a further rise in Sp^2^–C and a partial recovery of Sp^3^–C, consistent with XRD and Raman findings. However, it should be noted that distinguishing Sp^2^ and Sp^3^ carbons purely from C 1s spectra in amorphous carbons is challenging due to subtle binding energy shifts and peak broadening; therefore, the observed changes are interpreted as qualitative trends. The O 1s spectra (Figure 5e) can be deconvoluted into three peaks located at 531 eV (C=O), 532.3 eV (O–C–O), and 533.4 eV (O–C=O). It is noteworthy that the oxygen-containing functional groups in COF300&1300 and COF300&1300@C are richer than those in COF1300, suggesting that they can provide additional active sites for sodium ions, thereby enhancing the plateau capacity.

### 3.2. Electrochemical Performance and Sodium Storage Mechanism

**Figure 6:**
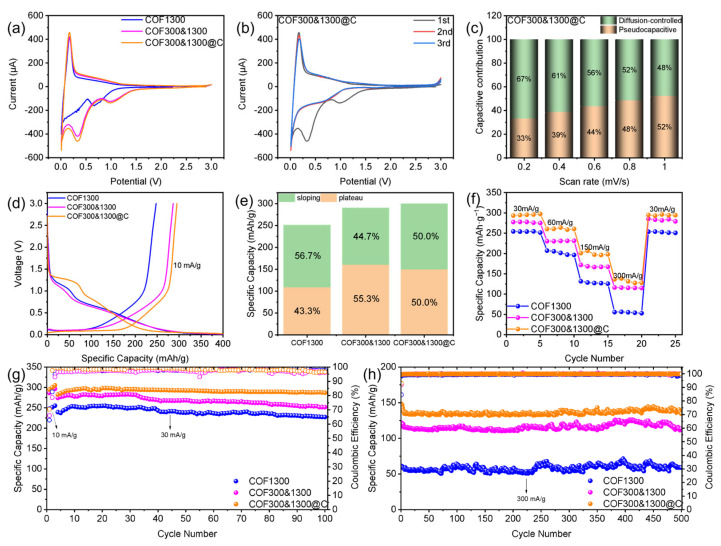
Electrochemical performance: (**a**) first CV curves of COF1300, COF300&1300 and COF300&1300@C at the scan rate of 0.1 mV s^−1^, (**b**) CV curves of COF300&1300@C at the scan rate of 0.1 mV s^−1^; (**c**) capacity contribution from diffusion-controlled and pseudocapacitive processes at different scan rates, (**d**) first cycle charge/discharge profiles at 10 mA g^−1^, (**e**) distribution of slope and plateau capacities in the second cycle, (**f**) rate capability, (**g**) cycling performance at 30 mA g^−1^, and (**h**) long-term cycling at 300 mA g^−1^ (1C).

A series of electrochemical tests were carried out to investigate the impact of pre-oxidation and carbon coating on the sodium storage performance. Figure 6a,b and Figure S2 show the cyclic voltammetry of the three materials at a scan rate of 0.1 mV s^−1^. All samples display a pair of reduction/oxidation peaks within the 0.01–0.2 V range, corresponding to the reversible insertion and removal of sodium ions among carbon layers. During the first cycle, they all exhibit a reduction peak around 0.7–1 V, then disappear in subsequent cycles, which is mainly associated with the formation of solid electrolyte interphase (SEI) during the initial cycle, with the SEI becoming increasingly stable in subsequent cycles. Specifically, COF300&1300 and COF300&1300@C exhibit an additional peak around 0.3 V, which may be attributed to the irreversible reaction (C=O + Na^+^ + e^−^→C–O–Na) [45]. Notably, COF300&1300 and COF300&1300@C exhibit a broader enclosed area within the low-voltage region of 0.01–0.4 V, indicating that they possess higher plateau capacity. Although an irreversible peak around 0.3 V is observed, subsequent cycles show stabilization, indicating that the dominant contribution in the 0.01–0.4 V range arises from reversible Na^+^ filling rather than continuous electrolyte decomposition. Additionally, the electrochemical reaction mechanism was investigated by cyclic voltammetry testing at 0.2–1 mV s^−1^ (Figure S3). The correlation between peak current ‘I’ and scan rate ‘v’ follows the equation I = av^b^, which serves to differentiate diffusion-controlled from surface-controlled processes. When b ≈ 0.5, the process is diffusion-controlled, corresponding to the insertion/extraction of sodium ions between graphite layers, whereas b ≈ 1 indicates a pseudocapacitive mechanism dominated by surface adsorption [46,47]. As summarized in Figure S4, during discharging, the b-values of all materials approach 1, indicating that pseudocapacitive mechanisms are predominant; during charging, the b-values approach 0.5, suggesting a shift toward diffusion-controlled intercalation. Regardless of whether it is discharging or charging, COF300&1300@C exhibits the highest b-value. This can be ascribed to the carbon coating, which reduces excessive surface area while regulating pore architecture, thereby stabilizing the interface and enabling faster Na^+^ transport. The capacitance contribution is quantitatively analyzed using the formula I = k_1_v + k_2_v^1/2^, where ‘k_1_v’ corresponds to the pseudocapacitive current and ‘k_2_v^1/2^’ reflects the diffusion-controlled process [48,49]. Figure S5 illustrates the pseudocapacitive and diffusion-controlled contributions of three materials at a 1 mV s^−1^ scan rate. All exhibit diffusion-controlled capacitance as the dominant mechanism, albeit with slight variations: The pseudocapacitive contribution of COF1300 is the largest, accounting for approximately 66%, indicating its strong ability to store charge through surface capacitance effects and higher sloping capacity. The pseudocapacitive contributions of COF300&1300 and COF300&1300@C are reduced to 54% and 52%, respectively, suggesting their relatively stronger ion diffusion capabilities during charging and discharging processes, and their higher plateau capacity. As illustrated in Figure 6c and Figure S6, the pseudocapacitive contribution rises as the scan rate increases across all samples. At elevated scan rates, the diffusion of Na^+^ into the bulk phase becomes insufficient, thereby increasing the relative dominance of surface-governed pseudocapacitive processes. Notably, for COF300&1300@C, its pseudocapacitive contribution ratio rises from 33% at 0.2 mV/s to 52% at 1 mV s^−1^.

The first cycle charge/discharge profiles of COF1300, COF300&1300, and COF300&1300@C at 10 mA g^−1^ are presented in Figure 6d. The reversible capacities are determined to be 248, 286, and 295 mAh g^−1^, with corresponding initial coulombic efficiencies (ICE) of 63%, 66%, and 70%, respectively. The higher ICE of COF300&1300 can be ascribed to the more uniform surface formed by pre-oxidation, which results in a smaller BET surface area, and thus fewer side reactions during the initial charge/discharge cycle. COF300&1300@C’s highest ICE is attributed not only to its pre-oxidation effect but also to the optimized microporous structure formed by carbon coating. This structure reduces SEI formation and electrolyte consumption by restricting solvent molecules from entering the pore walls [50]. Meanwhile, the pyrolytic carbon derived from acetylene can form a uniform coating layer on the HC surface, converting open pores into closed pores, thereby reducing the specific surface area and further improving ICE. The second cycle charging curves obtained at 10 mA g^−1^ are shown in Figure S7, while the proportion of sloping and plateau capacities are summarized in Figure 6e. Both COF300&1300 and COF300&1300@C show higher plateau capacity and higher sloping capacity than COF1300, consistent with the results of the capacitance contribution ratio at different scanning rates (Figure 6c and Figure S6). During the pre-oxidation process, oxygen-containing functional groups are introduced onto the surface of the hard carbon. During subsequent high-temperature carbonization, these groups decompose to release CO/CO_2_, inducing localized bending of the carbon layer and increasing the interlayer spacing, thereby enhancing the plateau capacity. At the same time, the locally bent carbon layer tends to form closed pores, further increasing the plateau capacity. Acetylene CVD treatment further enhances both plateau and sloping capacities: first, Sp^3^–C with C–H bonds and unstable functional groups is more prone to bond breaking, generating dangling bonds associated with free radicals and thereby inducing intrinsic defects such as edges, which improve the sloping capacity; second, defects such as pentagons and heptagons disrupt the integrity of the Sp^2^–C conjugated network, causing carbon layers to bend and enlarging the interlayer spacing, thus boosting the plateau capacity; finally, pyrolytic carbon from acetylene forms a uniform coating layer on the HC surface, converting open pores into closed pores. This process increases the number of closed pores and further enhances the plateau capacity.

The C-rate performance of HC was assessed by galvanostatic charge/discharge measurements conducted at current densities ranging from 30 to 300 mA g^−1^. As shown in Figure 6f, COF1300 delivers reversible capacities of 255, 205, 131 and 56 mAh g^−1^ at 30, 60, 150, and 300 mA g^−1^, respectively. Under the same conditions, COF300&1300 exhibits capacities of 278, 231, 172, and 116 mAh g^−1^, while COF300&1300@C demonstrates superior performance with capacities of 297, 264, 197, and 132 mAh g^−1^. Overall, COF300&1300@C exhibits the highest reversible specific capacity across all current densities and shows excellent capacity recovery upon decreasing the current density, thus delivering the best rate capability. The reasons are twofold: the presence of closed pores within the HC shortens the diffusion path, thereby strengthening bulk Na^+^ diffusion; meanwhile, the enlarged interlayer spacing lowers the diffusion barrier for Na^+^ migration.

The cycle performance was tested at a current density of 30 mA g^−1^ (Figure 6g). Due to its smaller interlayer spacing, COF1300 undergoes a capacity decline to 227 mAh g^−1^ after 100 cycles, corresponding to a retention rate of 89%. COF300&1300 delivers a high reversible capacity of 282 mAh g^−1^ and maintains 94% capacity retention after 100 cycles. Notably, COF300&1300@C exhibits optimal cycle stability due to its optimized pore architecture, featuring a high reversible specific capacity of 298 mAh g^−1^ and a capacity retention rate of 96% after 100 cycles. Additionally, COF300&1300@C exhibits significantly improved cycle stability compared to COF1300 and COF300&1300 at a higher current density of 300 mA g^−1^, maintaining a capacity retention rate of 97% after 500 cycles.

**Figure 7:**
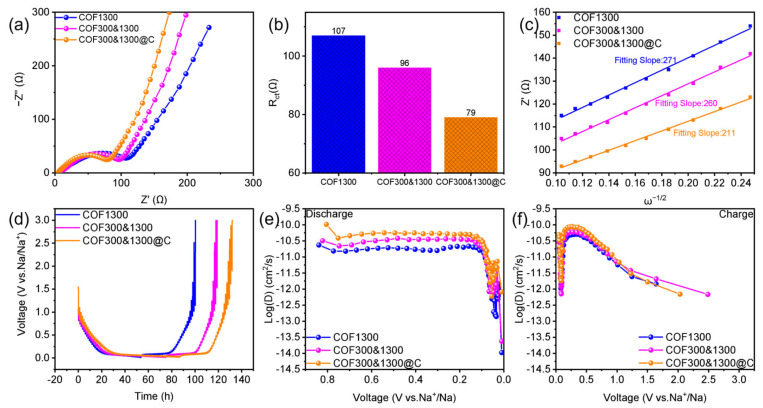
Electrochemical kinetics: (**a**) Nyquist plots from EIS, (**b**) corresponding R_ct_ values, (**c**) linear fits between Z’ and ω^−1/2^ for and Warburg coefficient determination, and (**d**–**f**) GITT profiles and calculated Na^+^ diffusion coefficients (D_Na_^+^) during discharge/charge.

The EIS spectra of COF1300, COF300&1300, and COF300&1300@C are shown in Figure 7a. The R_ct_ value for COF300&1300@C is 79 Ω, lower than that of COF1300 (107 Ω) and COF300&1300 (96 Ω). It can be attributed to at least two synergistic factors: Firstly, the uniform, conductive pyrolytic carbon coating creates a more efficient electrical percolation network on the particle surface, reducing the overall electronic resistance of the electrode. Secondly, the sealed surface and reduced SSA lead to a more stable and thinner SEI layer, facilitating a lower resistance for Na^+^ desolvation and charge transfer at the solid–liquid interface. In addition, the low-frequency linear region of COF300&1300 and COF300&1300@C exhibits steeper slopes compared with that of COF1300, suggesting faster Na^+^ diffusion kinetics in the modified materials. The Na^+^ diffusion coefficient can be determined using the equation D = (R^2^T^2^)/(2A^2^n^4^F^4^c^2^σ^2^) [51]. However, the standard semi-infinite linear diffusion model (Warburg impedance) assumes that ion diffusion is uniform, continuous, and infinitely extendable; yet, closed pores and complex pore networks introduce finite diffusion paths, reflective boundaries, and multiscale transport resistances, thereby limiting its applicability to complex closed-pore microstructures. The Na^+^ diffusion coefficients for COF1300, COF300&1300, and COF300&1300@C are 2.740 × 10^−13^ cm^2^ s^−1^, 2.977 × 10^−13^ cm^2^ s^−1^, and 4.515 × 10^−13^ cm^2^ s^−1^, as listed in Table 1. In particular, COF300&1300@C exhibits the fastest Na^+^ diffusion rate.

The Na^+^ diffusion coefficients were quantitatively determined using GITT, as shown in Figure 7d–f. The diffusion coefficients of all three materials exhibit similar trends with changing voltage. During the discharge process, the Na^+^ diffusion coefficient is relatively high in the sloping (high-voltage) region, reflecting surface-controlled processes such as adsorption at defect sites and interactions with residual functional groups, which involve short diffusion paths and faster kinetics. By contrast, in the plateau (low-voltage) region, Na^+^ storage occurs mainly through intercalation into carbon layers and filling of closed pores, processes that demand higher energy barriers and longer diffusion paths, resulting in a pronounced decrease in diffusion coefficients. On average, COF300&1300@C exhibits the highest Na^+^ diffusion coefficient, followed by COF300&1300, with COF1300 showing the lowest values, which is consistent with their rate performance trends. The GITT results prove that the enlarged interlayer spacing lowers the diffusion barrier of Na^+^. The optimized pore structure formed by the introduction of oxygen-functional groups and C-coating further lowers the diffusion barrier, facilitating Na^+^ diffusion and thereby enhancing electron transport rates within HC materials.

### 3.3. Comparative Analysis and Synergistic Mechanism

Table 2 compares the coffee grounds-based hard carbon prepared in this study with that prepared by other researchers. Some materials exhibit superior performance in terms of capacity or initial coulombic efficiency (ICE), primarily due to the modification method involving elemental doping. In this work, COF300&1300@C achieves an excellent balance of moderate capacity (298 mAh g^−1^), good ICE (70%), ultralow SSA (2.1 m^2^ g^−1^), and outstanding long-term cyclability at a high rate—a combination rarely reported. The proposed synergistic mechanism is summarized in Figure 1. Pre-oxidation serves as the *structural director*: Oxygen-containing functional groups promote cross-linking, suppress random defect formation, guide the development of a more ordered carbon skeleton with expanded interlayers, and create a preliminary pore network. CVD treatment acts as the *structure stabilizer and modifier*: The carbon coating forms a conductive layer that effectively seals external open pores, leading to a significant reduction in specific surface area. In addition, the coating regulates the pore architecture and helps stabilize closed-pore domains, thereby mitigating side reactions and improving the structural robustness of the carbon framework during electrochemical cycling.

## 4. Conclusions

In summary, we utilized coffee grounds as biomass feedstock and employed a strategy combining simple pre-carbonization with acetylene CVD to prepare coffee grounds-based hard carbon materials with optimal pore structures. The synergistic effect of the two processes simultaneously adjusted the pore structure and increased the interlayer spacing, resulting in a low specific surface area (2.1 m^2^g^−1^). It also increased the d_(002)_ spacing (0.391 nm) and optimized the distribution of closed pores. These structural features directly translate into enhanced electrochemical performance, including a high ICE of 70% and a high reversible specific capacity of 298 mAh g^−1^ at a current density of 30 mA g^−1^, with a capacity retention rate of 96% after 100 cycles. It further demonstrates enhanced rate performance and cycling stability, achieving a high reversible specific capacity of 132 mAh g^−1^ at a current density of 300 mA g^−1^, with a capacity retention rate of 97% after 500 cycles. Our work elucidates the fundamental principles of closed-pore engineering through synergistic treatments and provides a versatile and scalable pathway for transforming abundant biomass waste into high-performance anode materials for sustainable sodium-ion batteries.

## Figures and Tables

**Table 1:** Charge transfer impedance R_ct_, Warburg impedance coefficient σ and sodium-ion diffusion coefficient D_Na_^+^ of COF1300, COF300&1300 and COF300&1300@C.

	R_ct_ (Ω)	σ	D_Na_^+^ (cm^2^ s^−1^)
COF1300	107	271	2.740 × 10^−13^
COF300&1300	96	260	2.977 × 10^−13^
COF300&1300@C	79	211	4.515 × 10^−13^

**Table 2:** Comparison table of electrochemical properties of HC-CVD@C and other coffee ground-based hard carbons.

Raw Materials	Synthetic Temperature (℃)	ICE (%)	Cycle Ability (mAh g^−1^)
Coffee ground (Mg-doping) [52]	1300	80	340 at 30 mA g^−1^
Coffee ground [53]	1500	66	254 at 30 mA g^−1^
Coffee ground (KOH activation) [54]	900	61	223 at 50 mA g^−1^
Coffee ground (P-doping) [55]	1300	83	341 at 20 mA g^−1^
This work	1300	70	298 at 30 mA g^−1^

## Data Availability

The data supporting this article have been included as part of the Supplementary Information (SI). Supplementary Information is available. Data will be made available on request.

## Supplementary Materials

The following supporting information can be downloaded at: https://www.mdpi.com/article/10.3390/ma19122495/s1.
