# Supplementary material for: Synergistic Pre-Oxidation and CVD Engineering for Precise Closed-Pore Construction in Coffee Grounds-Derived Hard Carbon Anodes for High-Performance Sodium-Ion Batteries
Source: Materials (Basel). 2026 Jun 10;19(12):2495. doi: 10.3390/ma19122495 (PMC13302419; doi:10.3390/ma19122495)
Supplement: Supplementary file 1 [file materials-19-02495-s001.zip › materials-4342541-supplementary.pdf]

Supplementary Information

Synergistic Pre-carbonization and CVD Engineering of Coffee grounds-based Hard Carbon for Sodium-ion Batteries

Xinjie Sun[a], Hui Yang[a*]

[a] College of Materials Science and Engineering, Nanjing Tech University, Nanjing 211816, Jiangsu, China.

*E- mail address:

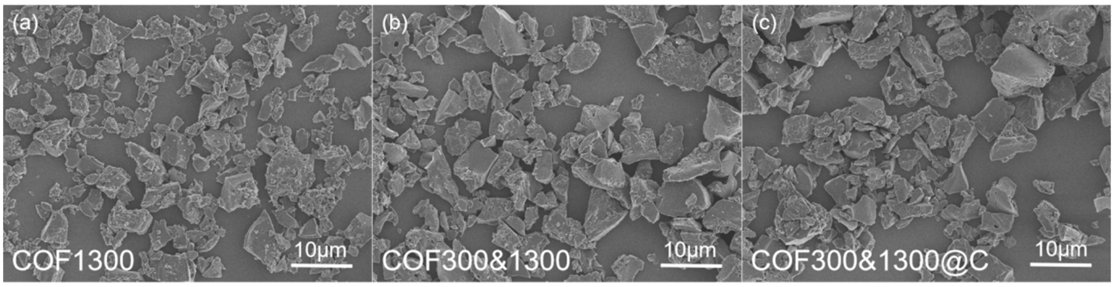

Fig. S1. Low-magnification SEM images of (a) COF1300, (b) COF300&1300, and (c) COF300&1300@C.

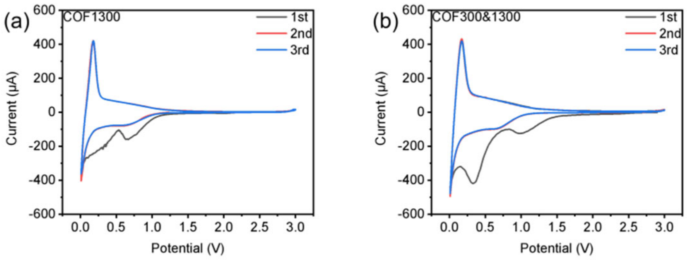

Fig. S2. CV curves of (a) COF1300 and (b) COF300&1300 at the scan rate of 0.1 mV s^{-1}.

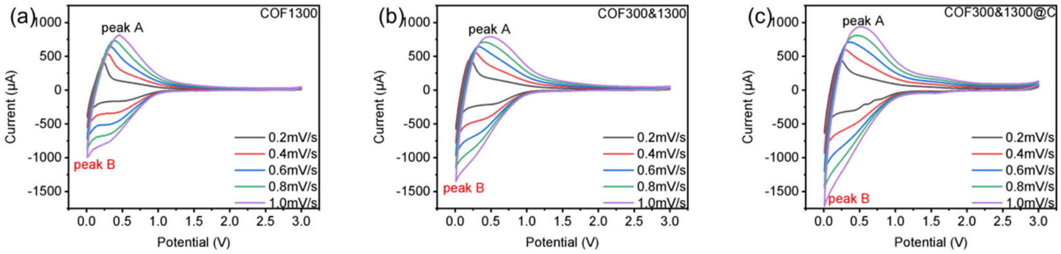

Fig. S3. CV curves of (a) COF1300, (b) COF300&1300, and (c) COF300&1300@C at scan rates of 0.2-1 mV s^{-1}

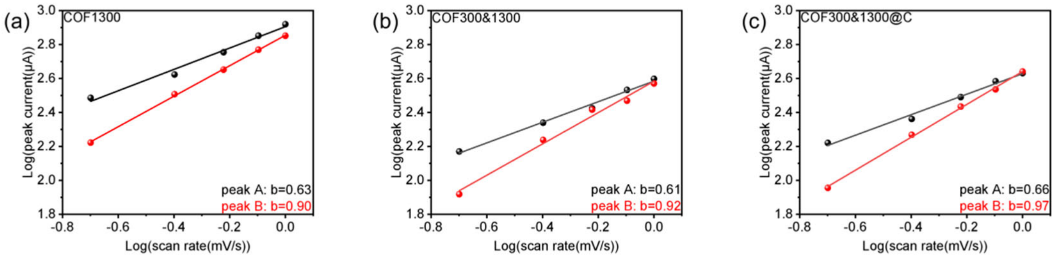

Fig. S4. b-value of (a) COF1300, (b) COF300&1300, and (c) COF300&1300@C using the relationship between the peak currents and the square root of scan rates.

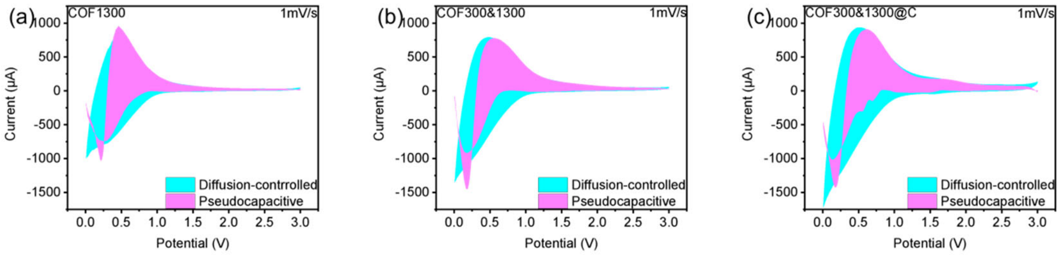

Fig. S5. Pseudocapacitive contribution and diffusion-controlled contribution of (a) COF1300, (b) COF300&1300 and (c) COF300&1300@C at 1 mV s^{-1}.

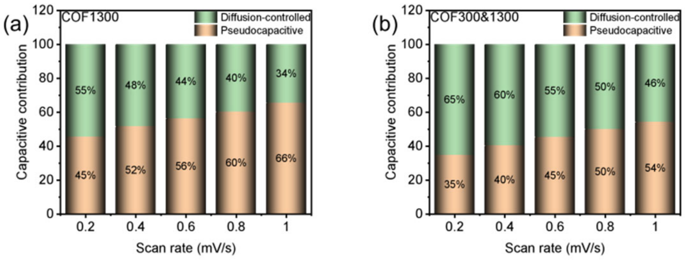

Fig. S6. Capacity contribution from diffusion-controlled and pseudocapacitive at scan rates of 0.2-1 mV s^{-1} for (a) COF1300 and (b) COF300&1300.

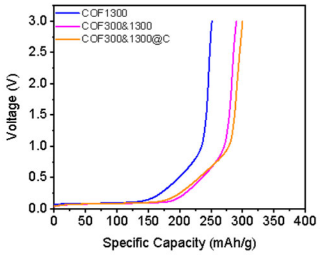

Fig. S7. Charging curves of (a) COF1300, (b) COF300&1300 and (c) COF300&1300@C during the second cycle at 10 mA g^{-1}.

Table S1. Hard carbon properties including: $d_{(002)}$, L_c, and L_a determined using XRD, I_D/I_G deduced using Raman spectroscopy, SSA and TPV obtained from N_2 adsorption-desorption, C, O and N amounts assessed using XPS.

Material	$d_{(002)}$ (Å)	L_c (Å)	L_a (Å)	I_D/I_G	SSA (m^2 g^{-1})	TPV (cm^3 g^{-1})	C (at%)	O (at%)	N (at%)
COF1300	3.73	11.67	17.87	1.58	37.4	0.0208	93.02	5.56	1.42
COF300&1300	3.79	11.94	20.24	1.41	4.5	0.0108	87.89	10.53	1.57
COF300&1300@C	3.91	10.12	18.62	1.53	2.1	0.0032	93.07	6.22	0.71

$d_{(002)}$, interlayer space; L_c, crystallite size; L_a, crystallite length; SSA, specific surface area; TPV, total pore volume.

Table S2 Charge transfer impedance R_{ct}, Warburg impedance coefficient σ and sodium-ion diffusion coefficient D_{Na}^{+} of COF1300, COF300&1300 and COF300&1300@C

	**R_{ct}（Ω）**	**σ**	**D_{Na}^{+}（$cm^2\ s^{-1}$）**
COF1300	107	271	2.740×10^{-13}
COF300&1300	96	260	2.977×10^{-13}
COF300&1300@C	79	211	4.515×10^{-13}
